# Quality of Life in Rural Communities: Residents Living Near to Tembeling, Pahang and Muar Rivers, Malaysia

**DOI:** 10.1371/journal.pone.0150741

**Published:** 2016-03-14

**Authors:** Khairuddin Idris, Hayrol Azril Mohamed Shaffril, Sulaiman Md. Yassin, Asnarulkhadi Abu Samah, Azimi Hamzah, Bahaman Abu Samah

**Affiliations:** 1 Institute for Social Science Studies, Universiti Putra Malaysia, Selangor Malaysia; 2 Department of Professional Development and Continuing Education, Faculty of Educational Studies, Universiti Putra Malaysia, Selangor, Malaysia; 3 Department of Social and Development Science, Faculty of Human Ecology, Universiti Putra Malaysia, Selangor, Malaysia; TNO, NETHERLANDS

## Abstract

The main aim of this study is to identify the quality of life (QoL) among communities residing near the Tembeling, Pahang and Muar Rivers in Malaysia. This quantitative study used a constructed questionnaire as main tool to collect data on the QoL of river communities. A total of 240 villagers were selected as respondents. The results indicated that the dimensions of settlement, safety, involvement and social relationships, as well as education scored highest, while dimensions of physical environment, financial and job security yielded moderate scores. Dimensions of infrastructure facilities yielded a low mean score. Recommendations are provided, in the hope that our results may be useful for strategies that could enhance QoL of these river communities.

## Introduction

Malaysia is aiming to be a developed country by 2020. To achieve this, several efforts have been placed in order to accelerate the country’s development. A country is not rated as developed by its economic achievements and rapid progress of development alone, but, the quality of life (QoL) and well-being of its people play also a significant role. Developed countries are characterized by higher incomes, better educational achievements, better public health and higher life expectancy for example. One of the biggest challenges for the Malaysian government to realize their aim is to ensure that no gap exists in QoL between various groups and communities, in particular between urban and rural communities. For those living in remote areas such as the indigenous river communities, challenges arise as they live isolated and are especially vulnerable to floods. Therefore, the government needs to ensure that these river communities have a QoL comparable to their urban counterparts.

[1] accentuated the importance of measuring quality of life (QoL) to understand people’s overall satisfaction with their existence. Assessments of QoL offer researchers data pertaining to factors that would influence the social, environmental and economical aspects of a community. Measuring QOL is not an easy task, as it has hundreds of dimensions. Indeed, within the Malaysian scope alone, a number of studies have been conducted to measure QOL at a national and urban level [2–5, 6, 7]. Although these studies have successfully measured the QoL in Malaysia, few studies were done with regard to the QoL of rural communities, particularly river communities. This brought forth the present study’s main objective;to investigate the QoL of Tembeling, Pahang and Muar Rivers’ communities. This study is significant in several ways. First, it contributes to the body of knowledge pertaining to river communities’ QoL since river communities are one of the earliest human settlements among most localities. Previous studies on QoL in Malaysia were prone to objective measurements, this study however interposes new knowledge as it proffers subjective measurement of QoL—which is based on the community’s perception and not the calculation of secondary data [8]. Second, the present study’s output has confirmed there are more QoL aspects to be included and serves as a foundation for future studies on the river communities’ QoL. Third, the contribution to policy making as findings from the study could disclose facts on strengths and weaknesses regarding river communities’ QoL, which eventually could assist relevant parties in constructing the best strategies to enhance river communities’ QoL.

### Subjective Measurement vs Objective Measurement of QoL

QoL measurement is an important method for acquiring knowledge on the well-being of an individual or a society. As QOL covers a wide range of context, there are a number of QOL studies that apply several ways to measure QOL The objective measurement of QoL is based on a set of reported characteristics. It involves measurement of quantified indicators and ‘materialistic’ aspects such as income, expenses, assets and the ownership of goods and also ‘non-materialistic’ aspects such as health, social inclusion, education and others [9]. The subjective measurement of QoL on the other hand, relies more on self-reported data collected through a survey, in which individuals assess their current life based on a scaled attribute relating to QoL domains [10].

### Tembeling, Pahang and Muar Rivers and Its Relation to the River Community

The Tembeling River is the main tributary of Pahang River (S1 Fig). It is well-known among local and international tourists in relation to eco-tourism activities. Today, locals still rely on this river to conduct their socio-economic activities. In remote villages such as Bantal, Mat Daling and Kuala Sat Pagi, the Tembeling River is the only route that connects them to the outside world. In addition, few locals have been conducting aquaculture activities on Tembeling River, rearing fish species such as patin (*Pangasius sutchi*) and tilapia.

The Pahang River is the longest river in Peninsular Malaysia, which originates from Jelai River and Tembeling River as it flows 459 km along two states—Pahang and Negeri Sembilan—and completes its course at Kuala Pahang, Pekan, and Pahang (S1 Fig). Pahang River is renowned for its farmed patin (*Pangasius spp*), and Pahang is the country’s main supplier for this species [11]. Parts of Pahang River have been developed as a recreational areas for fish and shrimp netting, and the aborigines in remote areas still rely on Pahang River for their mobility.

The Muar River’s upper portion is located near Talang village, Tanjung Ipoh, Kuala Pilah, and its course ends at Muar, Johor (S2 Fig). In the old days, Muar River used to be the main route for traders to reach the East-Coast region of Peninsular Malaysia. The river flow from Serting to Bera in Pahang, and Jempol to Muar River allowed traders from the west coast to travel by water to the east-coast states such as Pahang, Terengganu and Kelantan. Via Penarikan portage the distance travelled was about 300 meters, the traders were able to pull their boats onto land with help from the locals, hence the name Penarikan, which means ‘Pulling’ in Malay. Today, the Muar River is no longer a main trading route. According to [8], locals now rely on theMuar River to fulfil their recreational activities such as fishing and camping.

### Malaysian QoL Dimensions (Yassin et al. 2011)

#### Housing

Housing is essential for basic needs in life and should be construed in any QoL measurement [5, 7, 12, 13, 14, 15]. [7] have confirmed that the rural communities in Malaysia are highly satisfied with QoL in terms of housing. One reason is due to Malaysian government’s continuous efforts to ensure that all Malaysians, especially the low and middle-income group, have the opportunity to buy their own houses by establishing affordable-housing programs such as *Program Perumahan Rakyat*, *Rumah Mampu Milik* and *Rumah Mesra Rakyat*. The program allows low-income households the opportunity to buy low-cost housing with access to electricity and water [5].

#### Physical environment

Malaysian communities who are surrounded by healthy physical environment have reported better QoL [7, 14]. [5] has confirmed that some QoL aspects such as physical environment, especially water and air quality, have increased. Furthermore, the Malaysian government has accentuated the value of forested lands, which led to the implementation of green programs such as Sustainable Forest Management, Central Forest Spine Project in Peninsular Malaysia, and Heart of Borneo in Sabah and Sarawak.

#### Safety

The enhancement of Malaysian’s QoL in safety is reflected by the decline in crime rate and it is one of the National Key Result Areas (NKRA) focused by Malaysian government. [5] claimed in their study that a decrease in crime rate of 712 crime cases per 100,000 people to 628 cases was reported in 2010. This reduction may have stemmed from anti-crime programs organized by authorities, such as installation of closed-circuit televisions, and continuous police patrolling in high-risk crime areas. Furthermore, community-based volunteer services to patrol residential areas, such as *Skim Rondaan Sukarela* and *Rakan Cops* have contributed to reducing the crime rate. [7] and [16] both supported findings from [17] by emphasizing the fact that a police presence and feeling safe from crime can greatly contribute towards a community’s QoL.

#### Involvement and social relationships

A community’s involvement and social relationships are linked to their level of QoL. Studies by [2], [4–5], [17], [18], [19], [20], [21] have all confirmed that social activities involving participation from resident associations and non-profit organizations have contributed towards a better QoL. Although [7] have confirmed that a community’s QoL improves when residents involve themselves in charity and environmental-awareness activities, their findings disclose several opportunities to strengthen the community’s involvement in politics and sports activities. [22] on the other hand, demonstrated that rural communities have benefited from strong social relationships and activities through *gotong-royong* and *merewang* (both practices refer to a collective act of helping out each other during big events among members of the community), while [23] supported this by confirming that people living in remote areas tend to have stronger social relationships.

#### Education

Globally, education has always been considered as an important contributor towards a better QoL [14, 16, 18, 20, 24, 25, 26] and similar scenario applies in Malaysia [2–5, 7, 15]. Education would ensue better literacy rate, higher enrolment rates, an improved teacher–student ratios for primary and secondary schools, and higher percentage of graduate teachers in schools. Currently, the literacy rate in Malaysia is 95%, with a total of 5.4 million pre-school, primary and secondary school students and approximately 0.4 million teachers. To stress the importance of childhood education, several pre-school programs have been strengthened by the PERMATA Programme that provides education for children under four years old. Tabika Kemas is another early-childhood education center, as of 2011, a total of 9,966 Tabika Kemas have been available across Malaysia, and a total of 150,000 children from suburbs and rural areas are expected to have access to early-childhood education by 2015 [5].

#### Financial and job security

Financial and job security is another important dimension that has been constantly measured in several QoL studies [6, 15, 17, 27, 28, 29–30]. [41] said that Malaysian’s QoL in terms of income and job security satisfaction should be considered based on [5]. QoL record related to income yielded a high score, and a number of potential explanations are provided. First, according to [31], the average household income in Malaysia was RM 5,000 per month, with majority of households fall into the RM 3,000–RM 3,999 income group. According to [31], the country’s labor force consists of 10.89 million people, which means that majority of Malaysians were employed. Acknowledging that financial and job security of a person plays vital role in enhancing QoL, the Malaysian government has placed efforts to further reduce unemployment rates in Malaysia by creating more job opportunities. In 2006, for example, a total of 834,675 job vacancies were available across several sectors [31].

#### Infrastructure facilities

Infrastructure facilities can also influence a community’s QoL [5, 7, 15]. Improving infrastructure facilities within rural and remote areas to further enhance QoL has always been a part of the Malaysian government’s initiatives, which later becomes a part of the established NKRA agenda. Over four years (2006–2009), a total of 1,419.26 km of new and repaired roads are made available in rural areas, plus a total of RM47 million investment to build new public buildings in 2010 [32].

### QoL: Malaysia vs Neighboring Countries

Generally, several studies have tried to rank QoL among countries around the world, including South East Asia (ASEAN) [9, 33–36] (S1 Table). Looking across past studies, the ranking of QoL between ASEAN countries namely, Singapore, Malaysia, Thailand, the Philippines and Indonesia have been consistently placed within top five.

Generally, dimensions such as social life, income, education and health are consistently measured for ASEAN citizens’ QoL. Looking at past studies, various QoL dimensions have emerged across these countries. Some neighboring countries are enjoying similar QoL dimensions as Malaysians. The Thais for example, are enjoying better QoL when it comes to social life attributed to personal domains of QoL such as spiritual life, family life and individual life [37–38]. Singaporeans on the other hand are having a better QOL regarding political and social environment, economic environment and socio-cultural environment [39–40]. Furthermore, Vietnamese yielded better QoL in physical health, social relationship, finance and economics, physical and social environment, psychological health, and religious practices [41], whereas Indonesians and Philippines yielded better QoL in housing, standard of living, household income, health, education, job, family life, neighbors, public safety, environmental condition, social welfare system, spiritual life, friendships, marriage and leisure [42].

## Methodology

The study requires an ethical declaration for the study as human subjects are involved and has been approved by Institute for Social Science Studies Ethical Committee Board.

Participants were provided with an explanation to acquire their consent before proceeding with the questionnaire. After consents were obtained, the subjects were free to accept or reject the invitation to be involved in the study. A written consent was not obtained due to time limitation, besides, a verbal consent was deemed as adequate. The participants’ consent were recorded in the questionnaire and the Ethical Committee Board for Research from Institute for Social Science Studies did not have any problem in approving this procedure.

Respondents aged between 15 to 17 years old had verbal consents from their parents prior to their participation in this study. The parents were free to accept or reject the involvement of their children in the study. A written consent was not obtained due to time limitation and a verbal consent was deemed as adequate by the Ethical Committee Board of research.

This quantitative study employed a cluster sampling procedure and managed to select a total of 240 villagers residing alongside Tembeling, Pahang and Muar Rivers. The respondent’s age were between 15 to 84 years old. Only one member per household was eligible as a respondent. Based on G-Power analysis, the study was able to gain an appropriate sample number. G Power analysis refers to the process of obtaining minimum number of samples for each analysis. Power can be understood as a chance that the test will detect a statistically significant difference or a relationship when such difference or relationship exists. Power also refers to the probability to reject a null hypothesis. It is generally agreed that power should be .80 or greater, that is 80% or bigger opportunity of finding a statistically significant difference or relationship when there is one. To answer the objectives, ANOVA, independent t-test and Pearson product moment correlation analyses were performed. Referring to G-power analysis, with an alpha value of .05 and a magnitude of power between .90–.95, the appropriate number of sample to run an independent t-test is 176, while the appropriate sample to run ANOVA is 232 and the appropriate sample to run a Pearson product-moment correlation is 191. Having a big sample size is not a problem as [43] emphasized that a bigger sample size will strengthen the reliability and validity of the instrument.

Four areas were selected for the data collection process based on simple random sampling. A list of areas along Tembeling, Pahang and Muar rivers were acquired and four areas were randomly chosen for data collection. The selected areas were Bantal village (Tembeling River), Gintong village (Pahang River), Jorak village (Muar River) and Langkap village (Muar River). The socio-economic information for the four areas is presented in S2 Table. Each area were represented by 60 respondents (60 respondents x 4 areas = 240 respondents). These 60 respondents were selected randomly based on the list provided by respective village leaders.

A questionnaire developed by [7] was used for data collection. The questionnaire was originally developed by [7] via assorted documents and literature analyses Initial pre-test indicated a Cronbach alpha value of .856. For the purpose of this study, minor modifications were made in the demographic section. The questionnaire consists of two parts, namely, demographics (nine questions) and dimensions of QoL (51 questions) (S3 Table). Details on items focused in each QoL dimensions also appears on S3 Table.

The demographics section have both open-ended and closed-ended questions. As this study is more on the subjective measurement of river community’s QoL, the respondents were given options via a five-point Likert scale ranging from 1 (strongly agree) to 5 (strongly disagree). The bigger the scale chosen by respondents, the higher their agreement on the questions asked–for example, if a respondent ‘strongly agrees’ with education, it denotes that they ‘strongly agree’ that educational quality is high. The 5 Likert scale was shown to the respondents using a flash card with different color schemes to denote different dimensions of QoL. Each color scheme has a different degree of darkness to differentiate levels of scale. The process was administered gradually and cautiously to ensure the quality of gathered data.

Although the questionnaire had already been used by [7], the researchers pre-tested it again at Pekan using 30 respondents to further strengthen the reliability of the instrument. The recent pre-test Cronbach’s alpha value was in line with [7], and exceeded the value of .700 recommended by [44]. A survey method was applied during actual data-collection process, the process was conducted by trained and experienced enumerators monitored by the research team. The actual data collection took almost six months to complete, from May 2013 until October 2013. Through descriptive and inferential analyses, the study obtained the general data of study.

## Results

### Demographic Factor

As presented in S4 Table, 54.2% of respondents were male (compared to 45.8% female). The mean age was 39.7 years, and the majority of respondents fall into the age group of >41 years (44.6%). Majority of respondents (74.6%) were Malay, while the rest were of aborigines and Chinese (25.4%). In terms of educational achievement, most of the respondents possessed a primary education, compared to 12.9% who possessed a tertiary level of education. As these respondents lived near the rivers, some (22.6%) were involved in agriculture-related income generating activities, while another 21.4% were self-employed. The mean income per month of RM 1,118.45 (approximately equal to USD 270) exceeds the poverty level set by Malaysia Economic Planning Unit, which was RM 720 (approximately equal to USD 173). However, some respondents (31.4%) still earned below RM 500 (roughly equivalent to USD 166). In conclusion, most of the respondents were long-time residents as the mean score for period of residing in an area was 31.51 years with 12.1% having lived in the village for 40 to 50 years. The distance between their residence to either Tembeling, Pahang or Muar Rivers was 841 meters, with 23.3% of them living within less than 200 meters from the rivers.

### Level of the QoL Dimensions Studied

S5 Table demonstrates each dimension studied. It was concluded that onlyfour dimensions yielded high mean scores while other three QoL dimensions only yielded moderate mean scores. Education yielded the highest mean score (M = 3.92), while involvement and social relationships yielded the second highest mean score (M = 3.91). Furthermore, the respondents have demonstrated importance of housing in relation to QoL as this dimension yielded high mean score (M = 3.89). Another dimension that yielded high mean score was safety. Three factors that yielded moderate level of mean scores were infrastructure facilities, financial and job security and physical environment.

### Comparisons between Selected Demographic Factors and QoL Dimensions

Consecutively, the study compared QoL based on gender, area and education. The main motivation of studying a variety of QoL by demographical factors is the inconsistencies of findings from past studies. Within the scope of gender, for example, studies by [17] and [20] have confirmed that males have better involvement and social relationship, while a contradictory finding was concluded by [45]. Furthermore, [26] have agreed that education is associated with a community’s QoL, the higher their level of education achievement, the better their QoL will be, similar result cannot be concluded for the study by [7]. Having these inconsistencies have driven to query, how is the situation in Malaysia? Are males better than females with respect to their QoL? Is QoL of the Jorak community better than Bantal community? Are higher educated people better in terms of QoL? These questions underscores the need for further investigation into the possible dimensions of QoL.

#### Housing

To determine any significant difference in the factor of housing between males and females, an independent t-test was conducted. The analysis has concluded that there was no significant difference between males and females in their QoL regarding housing (S6 Table).

Generally, housing is the dimension that yielded the highest mean score. Further analysis has confirmed that there was significant difference between the areas studied and QoL (housing) (F value (4,240) = 10.745, p < 0.05). The Post-hoc analysis confirmed the significant difference between respondents in Jorak, Gintong and Langkap, compared to respondents in Bantal (S7 Table).

Regarding educational achievement, it was confirmed that there was no significant difference between levels of education and QoL (housing) based on F value (4, 240) = 1.984, p < .005. Hence, educational attainment does not play a role with regards to housing in the consideration of QoL (S7 Table).

To investigate any relationship between housing with age and income, a Pearson product moment correlation was performed. The analysis found that income had a significant and positive relationship with QoL related to housing. However, a negligible relationship (r = .151) exits between these two factors (S8 Table).

#### Physical environment

An independent t-test was employed to identify any difference in physical environment factor between males and females. Based on the scores for males (M = 3.58, SD = .710) and females (M = 3.97, SD = .804; t (240) = 1.377, p = 0.170), there was no significant difference between genders in physical environment factor (S9 Table).

One way ANOVA was performed to determine the differences between areas studied in terms of physical environment. The results have demonstrated that there was significant difference in physical environment between areas (F value (4,240) = 4.951, p < 0.05). Post hoc analysis have further confirmed that there was significant difference between respondents in Langkap and respondents in Jorak (S10 Table).

Additional analysis was performed to investigate the possibility of educational achievement impact on QoL with respect to physical environment. According to F value (4,240) = 1.262, p > 0.05, all groups have similar level of QoL regarding physical environment, thus indicating no significant differences between the groups (S10 Table).

Further analysis examined relationship between housing with age and income. Results show that both factors (p = .764 for age and r = .769 for income) did not affect the respondents’ QoL regarding physical environment (S11 Table).

#### Safety

Safety is another QoL factor studied and it was found that there was no significant difference identified between genders. This is based on the result for males (M = 3.71, SD = .883) and females (M = 3.85, SD = .809; t (240) = 1.344, p = 0.180) (S12 Table).

Analysis for safety has demonstrated significant difference in safety dimension among the areas in the study(F value (4,240) = 22.877, p < 0.05). Post hoc analysis has revealed that there was significant difference between respondents in Langkap and those in Bantal (S13 Table).

Educational achievement analysis between the four areas based on safety show no significant difference (F value (4,240) = .889, p > 0.05). Understandably, having such result has confirmed that educational achievement did not influence QoL in safety (S13 Table).

For safety, it was confirmed that both factors- age and income did not have any influence on QoL regarding safety. The results further confirmed there were no significant relationship between QoL and age (p = .619) and QoL and income (p = .424) (S14 Table).

#### Involvement and social relationships

For involvement and social relationship, analysis has confirmed a significant difference between genders. Based on mean for males (M = 4.06, SD = .883) and mean for females (M = 3.74, SD = .809; t (240) = 3.488, p = 0.001), it was concluded that males have better QoL compared to females in terms of their involvement and social relationship (S15 Table).

Results have demonstrated that there were significant difference in terms of involvement and social relationships between the four studied areas (F value (4,240) = 17.489, p < 0.05). Post hoc analysis confirmed a significant difference between respondents in Jorak, Gintong and Bantal, compared to those in Langkap (S16 Table).

Further analysis was performed to look into educational achievement’s influence over respondents’ involvement and social relationship. Based on F value (4,240) = 9.105, p < 0.05, there was significant difference between the four studied groups. Post hoc analysis has concluded a significant difference between those who possessed tertiary and secondary school level of education to those who have never been to school (S16 Table).

Furthermore, results showed that age was not significantly related to involvement and social relationship, while income was significantly related to respondent’s involvement and social relationship. The resulted r = .232 had concluded a weak relationship between income and involvement and social relationship (S17 Table).

#### Education

Gender was found to have no bearing on respondents’ QoL regarding education, based on the mean scores; M = 3.94, SD = .739 for males and M = 3.89, SD = .843; t (240) = .512, p = 0.642 for females. Having such result has confirmed that regardless their gender, these respondents had equal level of QoL related to education (S18 Table).

Correspondingly, one way ANOVA was used to identify differences between four studied areas in relation to education. Results have confirmed a significant difference in education between areas (F value (4, 240) = .703, p > 0.05). Each area yielded high mean scores, and the scores have demonstrated that all river communities have benefited from the government’s education initiatives (S19 Table).

As expected, the factor of education have an influence on the respondents QOL related to education. Based on the resulted analysis, the F value (4,240) = 8.339, p < 0.05 has determined that there was a significant difference between the four groups studied. Further analysis using a post hoc test has confirmed that there was a significant difference between those who possessed tertiary, secondary school and primary school levels of education with those who never been to school (S19 Table).

Within the scope of age and income, the Pearson product moment correlation analysis showed p = .622 for age and p = .429 for income. Hence, there is no significant relationship between two factors with respondents’ QoL related to education (S20 Table).

#### Financial and job security

An independent t-test was employed to determine differences between males and females in financial and job security. Based on results, males (M = 3.62, SD = .946) and females (M = 3.35, SD = 1.14; t (240) = 4.957, p = 0.044) showed significant difference in terms of financial and job security. Males were seen to have better QoL in financial and job security compared to females (S21 Table).

In terms of financial and job security, results have shown significant differences in the four studied areas (F value (4,240) = 6.697, p < 0.05). The post hoc test has revealed significant difference between Gintong and Jorak, compared to Bantal and Langkap (S22 Table).

Similar analysis was done to investigate whether educational achievement have any influence on respondents’ QoL in terms of financial and job security. Analysis (F value (4,240) = 4.441, p < 0.05) shows that there exists a significant difference between the four studied groups. Post hoc analysis has revealed there was significant difference between those who possessed tertiary level education to those who have never been to school (S22 Table).

Analysis have shown that income has significant and positive relationship with financial and job security (p = .0001) and r = .467 revealed that income has moderate relationship with financial and job security. Age however, had recorded no significant relationship (S23 Table).

#### Infrastructure facilities

Regarding infrastructure facilities and QoL, gender did not play any role and based on M = 2.83, SD = .991 for males and females (M = 2.76, SD = 1.05; t (240) = 1.609, p = 0.619) (S24 Table).

Results have also confirmed significant differences in infrastructure facilities between the four areas studied (F value (4,240) = 19.836, p < 0.05). Respondents in Jorak, Gintong and Bantal—yielded moderate mean scores, while respondents in Langkap yielded low mean scores (S25 Table).

Similar analysis was done to detect significant difference between educational achievement and respondents’ QoL in terms of infrastructure facilities. Based on F value of (4,240) = 19.836, p < 0.05), there was significant difference between the four groups studied and thepost hoc test has confirmed significant difference between those who possessed primary school and secondary school levels of education to those who have never been to school (S25 Table).

Pearson product moment correlation has determined that age did not have any significant relationship with infrastructure facilities, while income was seen to have positive and significant relationship with respondents’ QoL in terms of infrastructure facilities. Moreover, the resulted r = .196 indicates a negligible relationship between income and infrastructure facilities (S26 Table).

## Discussion

In the following, further discussions were on the level of QoL aspects possessed by rural communities, significant results yielded by several factors on QoL and comparison between QoL rankings of the studied rural communities with the national QoL ranking.

### Level of QoL Aspects Possessed by Rural Community

In conclusion, four QoL dimensions have yielded high mean scores, with education yielding highest mean score (M = 3.92). This finding is in line with results found in international and local studies [2, 4–5, 7, 15, 19. 24, 25]. According to [7], rural communities possess better QoL in terms of education due to numerous government efforts in developing the education system within rural areas (e.g. increasing the number of schools and teachers). Involvement and social relationships (M = 3.91) yielded second highest mean scores. [23] accentuated that rural communities have good QoL in involvement and social relationships as they frequently organize *gotong-royong* and *merewang*. It is common for them to spend time in the morning or late in the evening with other villagers at coffee stalls or *wakaf* (small shelter usually available near coastal village or fishermen's jetty). The importance of housing is emphasized by respondents when it comes to QoL, as housing yielded high mean score (M = 3.89). This finding was in line with studies conducted by [5], [7], [12], [13], [15]. The result was attributed to housing scheme from the government under *Program Perumahan Rakyat*, *Rumah Mampu Milik* and *Rumah Mesra Rakyat* [5, 7]. Another dimension that yielded high mean score was safety, this result was in line with local and international findings [5, 7, 46]. Among potential causes of respondents’ high ranking QoL for safety was due to police presence and reduced crime rates [5, 7].

### Comparison between QoL Rankings of Rural Community Studied with National QoL Ranking

To provide better understanding on these communities’ QoL, the study intends to compare their QoL with average National’s QoL produced by [5]. Based on comparison between rankings of QoL components in present study and QoL at national level, several similarities and differences were identified between the two levels of QoL. Although a comparison was done, it should not be regarded as statistically significant because it was done descriptively. Intriguingly, QoL components for education yielded highest score on both levels, the rural communities and the national level (S5 Table). The national level claimed that high QoL in education was due to better literacy rates, enrolment rates, teacher-student ratios for primary and secondary schools and percentage of graduated teachers with diploma/degree in school [5], whereas present study claimed that high QoL in education was due to teaching quality, students’ discipline and school infrastructure.

On a national scale, transportation and communication were regarded as important to QoL, especially those who live in urban and sub-urban areas, to have their own transportation and access to systematic public transportation were considered as vitally important. Furthermore, higher income level and introduction to new car models have allowed people to buy their own car. In addition to transportation, Malaysians consider communication as an important contributor to better QoL. To possess advanced communication gadgets such as IPad and smart phones with data plan offers the community a better QoL. Nowadays, broadband infrastructure has geared up greater use for Internet services, this is reflected by the increase in Internet subscription from 71 to 167 per 1,000 population (EPU, 2011). Meanwhile, the present study on the other hand has regarded aspects of involvement and social relationship as another important aspect for QOL. Within rural scope, having such scenario was not surprising as [23], [22], and [7] have all confirmed that social relationships are intensified during social activities such as *gotong-royong* (mutual works) and *merewang* (mutual works during wedding festivities)are actively conducted by the locals.

### Significant difference between Selected Demographic Factors with QoL Aspects Studied

#### Housing

With regards to housing, the ANOVA test shows that areas and income yielded significant difference. Those who reside in Bantal have less QoL in housing compared to others. Although Bantal residents are surrounded by forest, due to inadequate facilities such as not having paved road, disposal areas, clean water and electric supplies, their QoL in terms of housing were reduced [7]. Other than housing, income also yielded significant result. Certainly, having a better income provides a better financial power for the respondents to have a better quality of life regarding housing [15].

#### Physical environment

The Post hoc analysis shows significant difference between respondents in Langkap and respondents in Jorak. This result was expected since Langkap is located within a forest reserve where there is low pollution and prohibition of any harmful activities for the environment, compared to Jorak which is located near to an industrial zone.

#### Safety

A post hoc analysis revealed significant difference between respondents in Langkap and those in Bantal. This finding is interesting since both Langkap and Bantal are located in forest reserves, however respondents in Langkap have better QoL in safety than respondents in Bantal. There are several possible reasons for this. First, although Langkap is located within a forest reserve, it is accessible by road. The safety and security teams (e.g. police, firemen, and ambulance staff) can access Langkap within 30 minutes from the nearest city. Nonetheless, Bantal may also be in a reserved forest area, but it is only accessible by boat. A trip to Bantal jettytakes almost three hours from the nearest city. Although Bantal is equipped with its own clinic and police station, the services offered are basic and not suitable in case of emergency situations. In addition, Bantal is vulnerable to floods, and the fastest way to send in rescue teams is via air.

#### Involvement and social relationship

Analyses have found that males have better QoL in involvement and social relationship compared to females. Several reasons can extracted. First, most of males are active in their interaction with external environment [17]. Second, most rural women in Malaysia are housewives, they tend to have a lot of commitments especially towards family, which limits their involvement in activities related to politics, sports and associations. Cultural and gender barriers are another obstacle within rural setting. Most women are housewives and they are expected to manage their family rather than be involved in other activities [19].

The Post hoc analysis shows significant difference between respondents in Jorak, Gintong and Bantal, compared to those in Langkap. Unexpectedly, this finding is new as previous studies have shown that aboriginals in Malaysia have strong social ties [23].

In another analysis performed, respondents with primary, secondary, and tertiary level of education yielded better QoL compared to those who have never been to school. Drawing on [21], education institutions have been recognized as social development agents in social systems. According to [21], the experiences in these institutions not only enhance their academic performance, but also play an important role in their social relationship development, both positively and negatively. This denotes that those who have chance to pursue their education in institutions (primary school, secondary school, college, university) have better chance to develop better social involvement and relationship.

Income is another factor that yielded significant result with QoL in terms of involvement and social relationship, however, it was identified to have weak relationships. It denotes that the higher the income they possessed, the less chance they have in enhancing QoL in terms of involvement and social relationship.

#### Education

Based on ANOVA, those who have never been to school yielded low QoL with regard to education compared to others. Certainly, such result is expected as higher level of education means better QoL can be achieved [26].

#### Financial and job security

Males yielded better QoL in financial and job security compared to females and this is in line with previous studies [27, 47]. This scenario was due to majority of females in this study were housewives and much of their financial needs were placed on their husbands. The housewife status also limits their chance to participate in any financial activities such as loan and investment.

The post hoc test revealed respondents in Gintong and Jorak yielded better mean scores in QoL compared to those in Bantal and Langkap. This was attributed by the fact that Gintong and Jorak respondents have access to several jobs. The community in Gintong for example, have access to Jerantut city, and those in Jorak have access to Bukit Pasir industrial zone. Bantal and Langkap on the other hand, are located in forest reserves and their residents rely on low income generating activity such as rubber tapping.

Educational achievement shows significant difference with QoL in terms of financial and job security to those with tertiary level of education, which yielded highest mean score while those who have never been to school yielded lowest mean score. [29] said to have better educational achievement provides better job opportunities, thus allow them to have a better income and job security.

Income yielded positive and significant relationship with QoL in terms of financial and job security. The relationship strength was only moderate. This scenario was expected since having better income places them in a better and stable financial position and such finding is in line with previous studies done by [17], [28], [30], [48] and [49] who confirmed there was significant relationship between income and better QoL.

#### Infrastructure facilities

The analyses shows significant difference between respondents in Jorak, Bantal and Gintong, compared to Langkap. Understandably, only Langkap is facing problems in terms of inadequate infrastructure facilities. Educational achievement and income yielded significant results with QoL in terms of infrastructure facilities, those who have never been to school and those with lesser income were found to yield lower score and such finding is in line with previous studies done by [15] and [7].

## Conclusion

Present article studied QoL among Tembeling, Pahang and Muar River’s communities. The analysis of this study confirmed that four QoL dimensions that yielded high mean scores were housing, safety, involvement, social relationships and education while lowest score was yielded by infrastructure facilities dimension.

Comparatively, this kind of findings have contributed to additional knowledge on Malaysian’s QoL, particularly rural communities. Although similar results have been found—education scored the highest in this study and for the national study, this subjective study found that teaching quality, students’ discipline and school infrastructure are the main contributors as opposed to study at a national level, which emphasized on literacy rates, enrolment rates, teacher-student ratios for primary and secondary schools and percentage of graduated teachers with diploma/degree in school. Furthermore, at the national level, it was found that people highly value facilities (e.g. transportation, communication) whereas within this study, rural communities were found to place much value on their social relationship.

It was concluded that demographical factors such as areas, income, gender and educational achievement are found to influence community’s QoL dimensions. Age was found to not influence any QoL dimensions and regardless of their age, rural communities enjoy similar level of QoL.

Compared to Jorak and Gintong villages, which are located near cities and industrial areas, Langkap and Bantal villages, which are more remote have comparatively yielded lower mean scores in several QoL dimensions. There was no significant difference regarding education between the four areas studied, which denotes strong probability that communities in these four areas have equally benefited from educational development in their areas.

To further enhance the rural communities’ QoL, it is recommended that the focus should be placed on efforts to further improve infrastructure facilities, while programs, strategies and plans on enhancing rural communities’ QoL should be focused on specific groups of lower income minority, females and lower education achievers.

The study have several limitations. First, it was conducted using only 240 respondents from four areas along Tembeling, Pahang and Muar Rivers., Consequently the results might be enriched if the number of respondents and areas are increased. Second, although there are QoL measurement instruments in existence, the present study relies on the instrument established by [7], which was specifically developed for measuring river communities’ QoL. In the future, it is suggested that additional aspects of QoL such as cultural, psychological, spiritual and politics be incorporated.

## Supporting Information

S1 Fig The flows of Tembeling River (in red) (source: Gazim et al. (2012). (DOCX)

S2 Fig The flows of Pahang River and Muar River (source: http://hikmahmelayu.blogspot.my/2015_04_01_archive.html). (DOCX)

S1 Table QoL ranking of selected ASEAN countries based on several studies. (DOCX)

S2 Table Areas studied. (DOCX)

S3 Table The questionnaire. (DOCX)

S4 Table Respondents’ demographic data. (DOCX)

S5 Table Level of mean score for each QoL dimension studied. (DOCX)

S6 Table Comparison between gender with QoL (housing). (DOCX)

S7 Table Comparison between areas and educational achievement with QoL (housing). (DOCX)

S8 Table Relationship between age and income with QoL (housing). (DOCX)

S9 Table Comparison between areas and educational achievement with QoL (physical environment). (DOCX)

S10 Table Comparison between areas and educational achievement with QoL (physical environment). (DOCX)

S11 Table Relationship between age and income with QoL (physical environment). (DOCX)

S12 Table Comparison between gender with QoL (safety). (DOCX)

S13 Table Comparison between areas and educational achievement with QoL (safety). (DOCX)

S14 Table Relationship between age and income with QoL (safety). (DOCX)

S15 Table Comparison between gender with QoL (involvement and social relationship). (DOCX)

S16 Table Comparison between areas and educational achievement with QoL (involvement and social relationship). (DOCX)

S17 Table Relationship between age and income with QoL (involvement and social relationship). (DOCX)

S18 Table Comparison between gender with QoL (education). (DOCX)

S19 Table Comparison between areas and educational achievement with QoL (education). (DOCX)

S20 Table Relationship between age and income with QoL (education). (DOCX)

S21 Table Comparison between gender with QoL (financial and job security). (DOCX)

S22 Table Comparison between areas and educational achievement with QoL (financial and job security). (DOCX)

S23 Table Relationship between age and income with QoL (financial and job security). (DOCX)

S24 Table Comparison between gender with QoL (infrastructure facilities). (DOCX)

S25 Table Comparison between areas and educational achievement with QoL (infrastructure facilities). (DOCX)

S26 Table Relationship between age and income with QoL (infrastructure facilities). (DOCX)
