# Supplementary figures and images for: Quality of Life in Rural Communities: Residents Living Near to Tembeling, Pahang and Muar Rivers, Malaysia
Source: PLoS One. 2016 Mar 14;11(3):e0150741. doi: 10.1371/journal.pone.0150741 (PMC4790859; doi:10.1371/journal.pone.0150741)

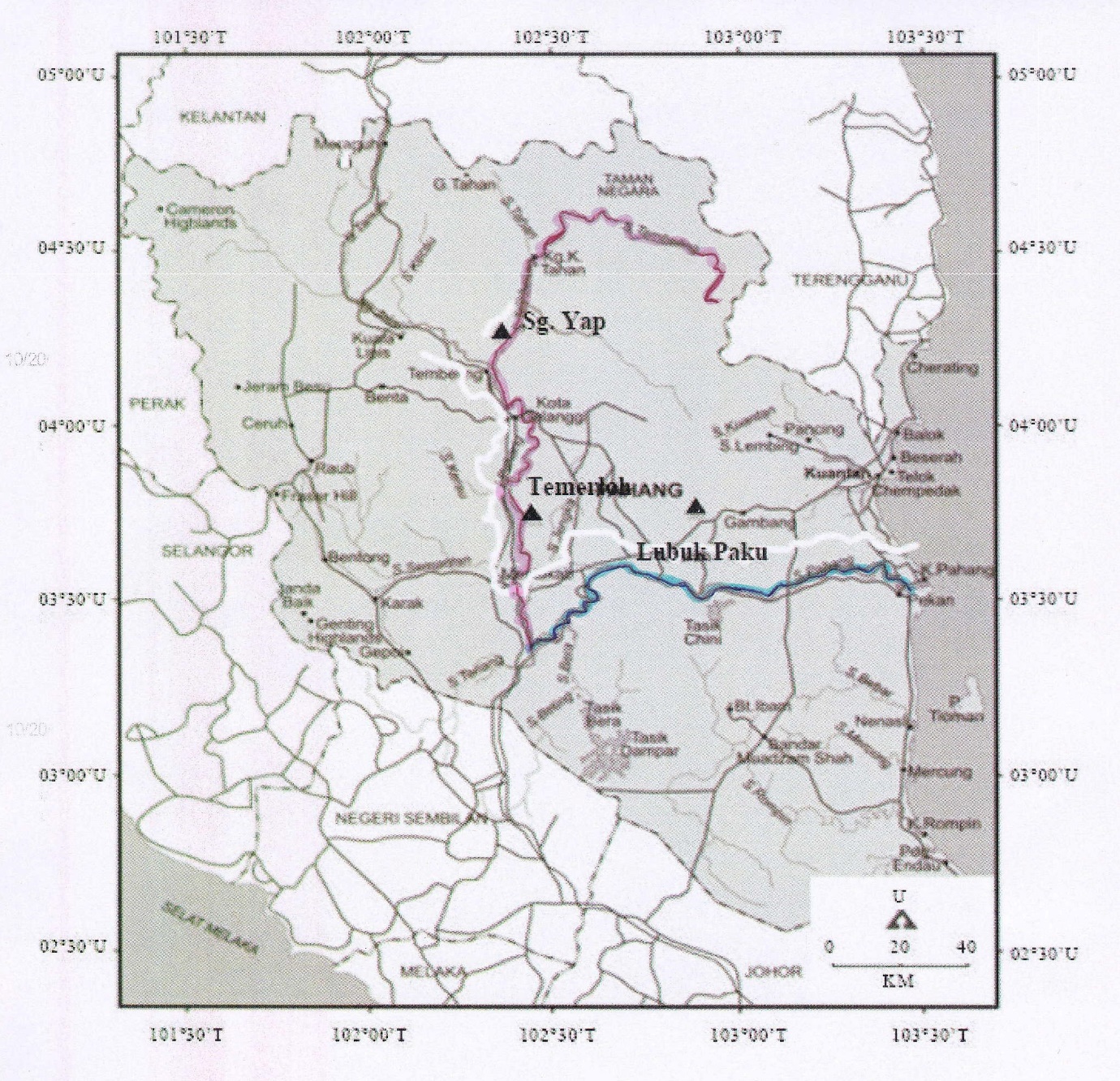


**S1 Fig. The flows of Tembeling River (in red) (source: Gazim et al. (2012)**

Supplement: S1 Fig — (DOCX) [file pone.0150741.s001.docx]
