# Supplementary material for: Quality of Life in Rural Communities: Residents Living Near to Tembeling, Pahang and Muar Rivers, Malaysia
Source: PLoS One. 2016 Mar 14;11(3):e0150741. doi: 10.1371/journal.pone.0150741 (PMC4790859; doi:10.1371/journal.pone.0150741)
Supplement: S2 Fig — (DOCX) [file pone.0150741.s002.docx]

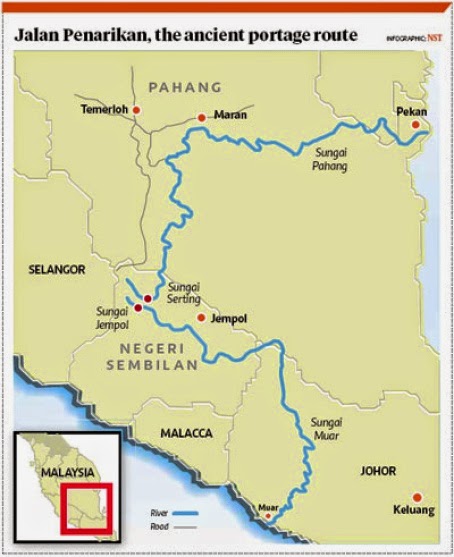


**S2 Fig. The flows of Pahang River (Sungai PahangBlue) and Muar River (Sungai MuarRed)** (source: <http://hikmahmelayu.blogspot.my/2015_04_01_archive.html>)
