# Supplementary material for: Quality of Life in Rural Communities: Residents Living Near to Tembeling, Pahang and Muar Rivers, Malaysia
Source: PLoS One. 2016 Mar 14;11(3):e0150741. doi: 10.1371/journal.pone.0150741 (PMC4790859; doi:10.1371/journal.pone.0150741)
Supplement: S1 Table — (DOCX) [file pone.0150741.s003.docx]

**S1 Table. QoL ranking of selected ASEAN countries based on several studies**

| Studies /QOL Ranking | Numbeo (2015) | The Economist (2013) | UNDP (2014) | IMD World Competitiveness (2013) | International living (2011) |
| --- | --- | --- | --- | --- | --- |
| First | Singapore | Singapore | Singapore | Singapore | Malaysia |
| Second | Malaysia | Malaysia | Brunei | Malaysia | Thailand |
| Third | Thailand | Thailand | Malaysia | Thailand | Singapore |
| Fourth | Philippines | Philippines | Thailand | Philippines | Brunei |
| Fifth | Cambodia | Vietnam | Indonesia | Indonesia | Indonesia |
