# Supplementary material for: Quality of Life in Rural Communities: Residents Living Near to Tembeling, Pahang and Muar Rivers, Malaysia
Source: PLoS One. 2016 Mar 14;11(3):e0150741. doi: 10.1371/journal.pone.0150741 (PMC4790859; doi:10.1371/journal.pone.0150741)
Supplement: S2 Table — (DOCX) [file pone.0150741.s004.docx]

**S2 Table. Areas studied**

| **Area** | **Main access** | **Main economic income** | **Distance to nearest city** | **Additional info** | **Population** | **Average**  **household income** | **Average age** | **Number of school** |
| --- | --- | --- | --- | --- | --- | --- | --- | --- |
| Gintong | Road | Agriculture activities, government sector | Roughly 14 km/15 minutes | Located near to Jerantut City  Vulnerable to floods  Adequate number of infrastructure facilities | 1200 | RM2,595 (roughly equal to USD800) | 41 | 1 school (less than 1 km) |
| Bantal | River | Agriculture activities | Roughly 70 km/3 hours by boat | Located at the Kuala Tahan National Park reserved forest areas  Vulnerable to floods  Adequate number of infrastructure facilities | 1200 | RM1,308 (roughy equal to USD400) | 38 |  |
| Jorak | Road | Industrial activities, agriculture activities | Roughly 20 km/15–25 minutes to the nearest city | Located near to the Bukit Pasir industry area  Vulnerable to floods  Adequate number of infrastructure facilities | 2000 | RM1,509  (roughly equal to USD500) | 45 | 1 school (less than 1 km) |
| Langkap | Road | Agriculture activities | Roughly 27 km/30–40 minutes to the nearest city | Located at the Pelangai reserved forest areas  Inadequate number of infrastructure facilities | 500 | RM559 (roughly equal to USD170) | 33 | 1 school (roughly around 15 km from Langkap village) |
