# Supplementary material for: Quality of Life in Rural Communities: Residents Living Near to Tembeling, Pahang and Muar Rivers, Malaysia
Source: PLoS One. 2016 Mar 14;11(3):e0150741. doi: 10.1371/journal.pone.0150741 (PMC4790859; doi:10.1371/journal.pone.0150741)
Supplement: S3 Table — (DOCX) [file pone.0150741.s005.docx]

**S3 Table. The Questionnaire**

| **Part** | **Number of questions** | **Focus of questions** |
| --- | --- | --- |
| Demographics | 9 | Questions related to demographic data |
|  |  |  |
| QOL |  |  |
| Housing | 11 | Spaces, number of rooms, suitability of locations, comfortability, electric supply, water supply, toilet facilities, drainage system, waste disposal services, roads |
| Physical environment | 7 | Waste disposal system, forest, hills, cleanliness of the river, cleanliness of the air, odor at the settlement area, noise at the settlement area |
| Safety | 7 | Safety, calmness, presence of police or security officers, community and properties safety, availability of the law, enforcement of the law, readiness to face disaster |
| Involvement and social relationships | 9 | Involvement in politic, involvement in charity, involvement in consumerism, involvement in environment conservation, involvement in sports, relationship with family members, neighbourhood relationship, satisfaction from leisure time and entertainment related activities. |
| Education | 5 | Education achievement, students discipline, school infrastructure, teaching quality, opportunity to pursue higher education |
| Financial and job security | 7 | Satisfaction on job status, relationship with colleagues, work environment, work security, financial status, access to loan services, financial affordability on medical services |
| Infrastructure facilities | 5 | Public transportation, worship facilities, recreational facilities, public toilet, government buildings |
| Total | 51 |  |
| **Overall total** | **60** |  |
