# Supplementary material for: Quality of Life in Rural Communities: Residents Living Near to Tembeling, Pahang and Muar Rivers, Malaysia
Source: PLoS One. 2016 Mar 14;11(3):e0150741. doi: 10.1371/journal.pone.0150741 (PMC4790859; doi:10.1371/journal.pone.0150741)
Supplement: S4 Table — (DOCX) [file pone.0150741.s006.docx]

**S4 Table. Respondents’ demographic data**

|  | **Frequency** | **Percentage** | **Mean** | **SD** |
| --- | --- | --- | --- | --- |
| Gender |  |  |  |  |
| Male | 130 | 54.2 |  |  |
| Female | 110 | 45.8 |  |  |
|  |  |  |  |  |
| Age (years) |  |  | 39.7 | 16.6 |
| 15–24 | 55 | 22.9 |  |  |
| 25–40 | 78 | 32.5 |  |  |
| >41 | 107 | 44.6 |  |  |
|  |  |  |  |  |
| Race |  |  |  |  |
| Malay | 179 | 74.6 |  |  |
| Aborigine | 59 | 24.6 |  |  |
| Chinese | 2 | 0.8 |  |  |
|  |  |  |  |  |
| Education achievement |  |  |  |  |
| Never been to school | 27 | 11.3 |  |  |
| Primary school | 85 | 35.4 |  |  |
| Secondary school | 97 | 40.4 |  |  |
| Tertiary level | 31 | 12.9 |  |  |
|  |  |  |  |  |
| Occupation |  |  |  |  |
| Government sector | 26 | 11.1 |  |  |
| Self-employed | 50 | 21.4 |  |  |
| Housewife/unemployed | 47 | 20.1 |  |  |
| Retiree | 10 | 4.3 |  |  |
| Agriculture-related | 53 | 22.6 |  |  |
| Student | 14 | 6.0 |  |  |
| Private sector | 21 | 9.0 |  |  |
| Businessman | 9 | 3.8 |  |  |
| Other | 4 | 1.7 |  |  |
|  |  |  |  |  |
| Income (RM) (N = 188) |  |  | RM1,118.46 | 984.99 |
| 500 and less | 59 | 31.4 |  |  |
| 501–1000 | 69 | 36.7 |  |  |
| 1001–1500 | 60 | 31.9 |  |  |
|  |  |  |  |  |
| Period living in area (years) |  |  | 31.51 | 20.49 |
| 10 and less | 46 | 19.2 |  |  |
| 11–20 | 41 | 17.1 |  |  |
| 21–30 | 43 | 17.9 |  |  |
| 31–40 | 28 | 11.7 |  |  |
| 41–50 | 29 | 12.1 |  |  |
|  |  |  |  |  |
| Distance to river (from residence) (meters) |  |  | 841.0 | 906.0 |
| 200 and less | 56 | 23.3 |  |  |
| 201–500 | 76 | 31.7 |  |  |
| 501–1000 | 66 | 27.5 |  |  |
| 1001 and more | 42 | 17.5 |  |  |
