# Supplementary material for: Quality of Life in Rural Communities: Residents Living Near to Tembeling, Pahang and Muar Rivers, Malaysia
Source: PLoS One. 2016 Mar 14;11(3):e0150741. doi: 10.1371/journal.pone.0150741 (PMC4790859; doi:10.1371/journal.pone.0150741)
Supplement: S5 Table — (DOCX) [file pone.0150741.s007.docx]

**S5 Table. Level of mean score for each QoL dimension studied**

|  | **Mean** | **SD** | **Median** |
| --- | --- | --- | --- |
| Housing | 3.89 | .76 | 4.00 |
| Physical environment | 3.67 | .80 | 3.57 |
| Safety | 3.77 | .85 | 3.86 |
| Involvement and social relationships | 3.91 | .74 | 4.00 |
| Education | 3.92 | .79 | 4.00 |
| Financial and job security | 3.49 | 1.05 | 3.86 |
| Infrastructure facilities | 2.80 | 1.01 | 3.00 |
