# Supplementary material for: Quality of Life in Rural Communities: Residents Living Near to Tembeling, Pahang and Muar Rivers, Malaysia
Source: PLoS One. 2016 Mar 14;11(3):e0150741. doi: 10.1371/journal.pone.0150741 (PMC4790859; doi:10.1371/journal.pone.0150741)
Supplement: S6 Table — (DOCX) [file pone.0150741.s008.docx]

**S6 Table. Comparison between gender with QoL (housing)**

| **Variables** | **Mean score** | **S.D** | ***t*** | ***p*** |
| --- | --- | --- | --- | --- |
| Gender |  |  | 1.377 | .170 |
| Male | 3.84 | .710 |  |  |
| Female | 3.97 | .804 |  |  |
