# Supplementary material for: Quality of Life in Rural Communities: Residents Living Near to Tembeling, Pahang and Muar Rivers, Malaysia
Source: PLoS One. 2016 Mar 14;11(3):e0150741. doi: 10.1371/journal.pone.0150741 (PMC4790859; doi:10.1371/journal.pone.0150741)
Supplement: S7 Table — (DOCX) [file pone.0150741.s009.docx]

**S7 Table. Comparison between areas and educational achievement with QoL (housing)**

| **Variables** | **Mean score** | **f** | **p** |
| --- | --- | --- | --- |
| **Areas** |  | **10.745** | **.0001** |
| Jorak | 4.14 |  |  |
| Bantal | 3.47 |  |  |
| Gintong | 3.92 |  |  |
| Langkap | 4.07 |  |  |
|  |  |  |  |
| **Education achievement** |  | **1.984** | **.117** |
| Never been to school | 3.6234 |  |  |
| Primary school | 3.8810 |  |  |
| Secondary school | 4.0066 |  |  |
| Tertiary level | 3.8475 |  |  |
