# Supplementary material for: Quality of Life in Rural Communities: Residents Living Near to Tembeling, Pahang and Muar Rivers, Malaysia
Source: PLoS One. 2016 Mar 14;11(3):e0150741. doi: 10.1371/journal.pone.0150741 (PMC4790859; doi:10.1371/journal.pone.0150741)
Supplement: S9 Table — (DOCX) [file pone.0150741.s011.docx]

**S9 Table. Comparison between areas and educational achievement with QoL (physical environment)**

| **Variables** | **Mean score** | **S.D** | ***t*** | ***p*** |
| --- | --- | --- | --- | --- |
| Gender |  |  | 1.911 | .057 |
| Male | 3.58 | .784 |  |  |
| Female | 3.78 | .813 |  |  |
