# Supplementary material for: Quality of Life in Rural Communities: Residents Living Near to Tembeling, Pahang and Muar Rivers, Malaysia
Source: PLoS One. 2016 Mar 14;11(3):e0150741. doi: 10.1371/journal.pone.0150741 (PMC4790859; doi:10.1371/journal.pone.0150741)
Supplement: S10 Table — (DOCX) [file pone.0150741.s012.docx]

**S10 Table. Comparison between areas and educational achievement with QoL (physical environment)**

| **Variables** | **Mean score** | **f** | **p** |
| --- | --- | --- | --- |
| **Areas** |  | **4.951** | **.002** |
| Jorak | 3.46 |  |  |
| Bantal | 3.60 |  |  |
| Gintong | 3.63 |  |  |
| Langkap | 3.99 |  |  |
|  |  |  |  |
| **Education achievement** |  | **1.262** | **.288** |
| Never been to school | 3.85 |  |  |
| Primary school | 3.74 |  |  |
| Secondary school | 3.58 |  |  |
| Tertiary level | 3.59 |  |  |
