# Supplementary material for: Quality of Life in Rural Communities: Residents Living Near to Tembeling, Pahang and Muar Rivers, Malaysia
Source: PLoS One. 2016 Mar 14;11(3):e0150741. doi: 10.1371/journal.pone.0150741 (PMC4790859; doi:10.1371/journal.pone.0150741)
Supplement: S11 Table — (DOCX) [file pone.0150741.s013.docx]

**S11 Table. Relationship between age and income with QoL (physical environment)**

| **Variables** | ***r*** | ***P*** |
| --- | --- | --- |
| Age | 0.20 | .764 |
| Income | 0.22 | .769 |
