# Supplementary material for: Quality of Life in Rural Communities: Residents Living Near to Tembeling, Pahang and Muar Rivers, Malaysia
Source: PLoS One. 2016 Mar 14;11(3):e0150741. doi: 10.1371/journal.pone.0150741 (PMC4790859; doi:10.1371/journal.pone.0150741)
Supplement: S22 Table — (DOCX) [file pone.0150741.s024.docx]

**S22 Table. Comparison between areas and educational achievement with QOL (financial and job security)**

| **Variables** | **Mean score** | **f** | **p** |
| --- | --- | --- | --- |
| **Areas** |  | **6.967** | **.0001** |
| Jorak | 3.73 |  |  |
| Bantal | 3.12 |  |  |
| Gintong | 3.84 |  |  |
| Langkap | 3.29 |  |  |
|  |  |  |  |
| **Education achievement** |  | **4.441** | **.005** |
| Never been to school | 3.03 |  |  |
| Primary school | 3.42 |  |  |
| Secondary school | 3.55 |  |  |
| Tertiary level | 3.97 |  |  |
