# Supplementary material for: Quality of Life in Rural Communities: Residents Living Near to Tembeling, Pahang and Muar Rivers, Malaysia
Source: PLoS One. 2016 Mar 14;11(3):e0150741. doi: 10.1371/journal.pone.0150741 (PMC4790859; doi:10.1371/journal.pone.0150741)
Supplement: S25 Table — (DOCX) [file pone.0150741.s027.docx]

**S25 Table. Comparison between areas and educational achievement with QoL (infrastructure facilities)**

| **Variables** | **Mean score** | **f** | **p** |
| --- | --- | --- | --- |
| **Areas** |  | **19.836** | **.0001** |
| Jorak | 2.93 |  |  |
| Bantal | 3.22 |  |  |
| Gintong | 3.02 |  |  |
| Langkap | 2.03 |  |  |
|  |  |  |  |
| **Education achievement** |  | **3.417** | **.018** |
| Never been to school | 2.26 |  |  |
| Primary school | 2.84 |  |  |
| Secondary school | 2.94 |  |  |
| Tertiary level | 2.74 |  |  |
